# Structures of Complexes of a Metal-independent Glycosyltransferase GT6 from *Bacteroides ovatus* with UDP-*N*-Acetylgalactosamine (UDP-GalNAc) and Its Hydrolysis Products*

**DOI:** 10.1074/jbc.M113.545384

**Published:** 2014-01-23

**Authors:** Tram T. K. Pham, Brittany Stinson, Nethaji Thiyagarajan, Michelle Lizotte-Waniewski, Keith Brew, K. Ravi Acharya

**Affiliations:** From the ‡ Department of Biology and Biochemistry, University of Bath, Claverton Down, Bath BA2 7AY, United Kingdom and; the § Department of Biomedical Science, Charles E. Schmidt College of Medicine, Florida Atlantic University, Boca Raton, Florida 33431

**Keywords:** Bacteria, Crystal Structure, Enzyme Mechanisms, Glycosyltransferases, X-ray Crystallography

## Abstract

Mammalian members of glycosyltransferase family 6 (GT6) of the CAZy database have a GT-A fold containing a conserved Asp-*X*-Asp (D*X*D) sequence that binds an essential metal cofactor. *Bacteroides ovatus* GT6a represents a GT6 clade found in more than 30 Gram-negative bacteria that is similar in sequence to the catalytic domains of mammalian GT6, but has an Asn^95^-Ala-Asn^97^ (N*X*N) sequence substituted for the D*X*D motif and metal-independent catalytic activity. Co-crystals of a low activity mutant of BoGT6a (E192Q) with UDP-GalNAc contained protein complexes with intact UDP-GalNAc and two forms with hydrolysis products (UDP plus GalNAc) representing an initial closed complex and later open form primed for product release. Two cationic residues near the C terminus of BoGT6a, Lys^231^ and Arg^243^, interact with the diphosphate moiety of UDP-GalNAc, but only Lys^231^ interacts with the UDP product and may function in leaving group stabilization. The amide group of Asn^95^, the first Asn of the N*X*N motif, interacts with the ribose moiety of the substrate. This metal-independent GT6 resembles its metal-dependent homologs in undergoing conformational changes on binding UDP-GalNAc that arise from structuring the C terminus to cover this substrate. It appears that in the GT6 family, the metal cofactor functions specifically in binding the UDP moiety in the donor substrate and transition state, actions that can be efficiently performed by components of the polypeptide chain.

## Teaser

**Background:** Bacterial homologues of human blood group synthases (glycosyltransferase family GT6) differ in being metal-independent.

**Results:** The structure has been determined of a GT6 from *Bacteroides ovatus* in a complex with the substrate UDP-GalNAc.

**Conclusion:** Interactions with the polypeptide replace substrate-metal interactions in metal-dependent mammalian homologues.

**Significance:** Metal independence in GT6 is attainable because the metal acts in substrate binding but not directly in catalysis.

## Introduction

Glycosyltransferases (GTs)^6^ are essential enzymes for the biosynthesis of polysaccharides and complex oligosaccharides that have vital roles in biological structures and processes including cellular interactions, matrix structures, signaling, pathogenesis, and immunity (1, 2). Their importance is emphasized by the substantial proportion (1–2%) of GT genes in the coding sequences of genomes from all domains of life (1, 2). Based on sequence and structural relationships, GTs are classified into about 90 divergent families in the CAZy database (3); they also form two groups based on whether the anomeric configuration of the transferred sugar is inverted or retained in the reaction product (3, 4). The members of each GT family are expected to have similar folds, and most known GT structures have one of the two predominant folds, designated GT-A and GT-B (4).

The members of GT family 6 (GT6) found in vertebrates are retaining enzymes that catalyze the formation of α-1,3 linkages between galactose or *N*-acetylgalactosamine and β-galactosyl or *N*-acetylgalactosaminyl residues in glycoconjugates. They are type II membrane proteins with small N-terminal cytosolic domains, a transmembrane helix, a stem, and the C-terminal catalytic domain with a GT-A fold. Although humans have four GT6 genes, α-1,3-galactosyltransferase (α3GT), isogloboside 3 synthase (iGb3S), histo-blood group A synthase (HBGA/B), and Forssman glycolipid synthase (Gb5S), the products of the α3GT, iGb3S, and Gb5S genes are catalytically inactive, and there are three alleles of the HBGA/B gene that generate individual variations in blood group structures (A, B, AB, or O) (5, 6). Adults have circulating natural antibodies against the glycan products of catalytically inactive GT6 (α-gal epitope, HBGA, HBGB, and Forssman glycolipid (Gb5)) (7) that help to provide immunity against enveloped viruses derived from host species that have active α3GT or Gb5 or from individuals with different histo-blood groups (5, 6). Interestingly, a new rare human blood group (Apae or FORS-1) has been found that is associated with a mutation in Gb5S that restores its catalytic activity (R296Q) (8). Because glycans function as receptors for pathogens, the loss or variation in surface glycans can reduce susceptibility to some toxins, bacteria, and viruses; for example the α-gal epitope is a receptor for *Clostridium difficile* enterotoxin A (9), Forssman glycolipid is a receptor for uropathogenic strains of *Escherichia coli* (10), and histo-blood group antigens are receptors for Norwalk virus (11).

**FIGURE 1.**
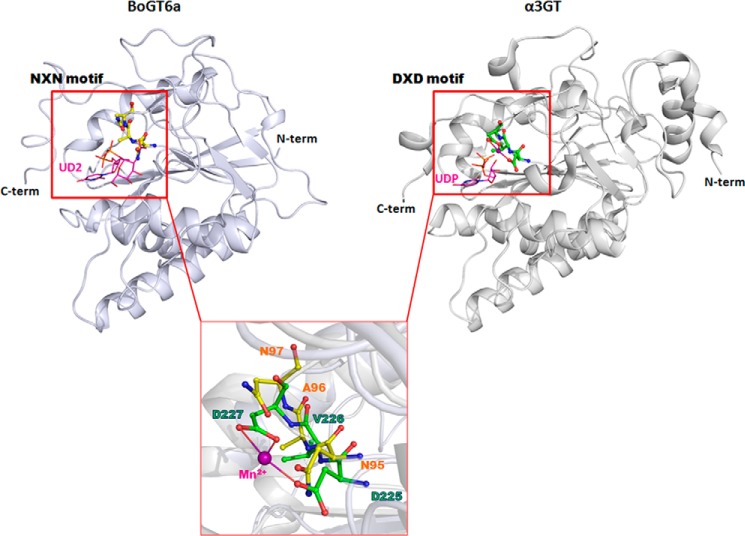
**Structural comparison of the metal independent BoGT6a (with the N*X*N motif in *silver*) and metal dependent bovine α3GT (with the D*X*D motif in *gray*).** The *inset* shows the details of the N*X*N motif (in *yellow*) and D*X*D (in *green*) motifs in BoGT6a (12) and α3GT (Protein Data Bank (PDB) code 1K4V (17)), respectively. The bound Mn^2+^ ion in α3GT is shown in purple sphere.

Although GT6 genes are ubiquitous in vertebrates, they have not been found in invertebrates, plants, fungi, yeast, or archaea. However, the expanding array of sequenced bacterial genomes has revealed genes encoding GT6 catalytic domains in 36 species of bacteria and two cyanophages. They are also found in two unicellular eukaryotic species. Many other GT6 genes from unidentified prokaryotic sources are present in the human gut and marine metagenomes (6, 12). With a single exception, the bacterial GT6 are from Gram-negative species and have substitutions of Asn for Asp in a highly conserved D*X*D motif that is responsible for binding a divalent metal ion cofactor in vertebrate GT6. A degenerate motif similar to D*X*D is present in all other GT-A fold GTs that depend on divalent metal ion cofactors for activity (4, 6). In previous studies we have functionally characterized one of the two GT6 from *Bacteroides ovatus*, BoGT6a, finding that it has a similar specificity to human HBGA synthase, catalyzing the transfer of GalNAc from UDP-GalNAc to 2′-fucosyl-lactose (FAL), and similar acceptors to produce A-antigen-like products (12, 13). Despite its close similarity in sequence to the catalytic domains of mammalian GT6, BoGT6a differs from them in not requiring divalent metal ions for activity (12) (Fig. 1). We also expressed one of two currently known bacterial GT6 that have a D*X*D motif, from *Parachlamydia acanthamoebae (*strain Hall's coccus), and found that it is dependent on divalent metal ions (Mg^2+^ or Mn^2+^) for activity, corroborating the link between this motif and metal dependence or independence (13).

Previously reported crystallographic structures of apo-BoGT6a (PDB code 4AYL) and its complex with the acceptor substrate FAL (PDB code 4AYJ) show that its interactions with the acceptor substrate involve residues that are mostly equivalent to those that bind the acceptor substrate in mammalian GT6 (13). To understand how this enzyme can bind its donor substrate, UDP-GalNAc, and be catalytically active in the absence of divalent metal ions, we have investigated its interaction with this substrate by structural studies of co-crystals. Because, like most other GTs, BoGT6a catalyzes the transfer of GalNAc from UDP-GalNAc to water (hydrolysis), albeit at a low rate in comparison with oligosaccharide acceptors, we used an enzyme mutant with Gln substituted for Glu^192^ that has attenuated activity (4 × 10^−5^ fold relative to wild type) (12). Even with this mutant, crystal forms were obtained containing some molecules with bound hydrolysis products together with others with intact substrate. Examination of these structures has revealed interactions and conformational changes associated with UDP-GalNAc binding and hydrolysis. Together with the previously described structure of the enzyme complex with FAL and mutational and binding studies (12, 13), the new structures indicate that substrate binding to BoGT6a is ordered, with UDP-GalNAc binding prior to acceptor, and have some implications regarding the catalytic mechanism and role of metals in the GT6 family.

## EXPERIMENTAL PROCEDURES

### 

#### 

##### Protein Expression, Purification, and Crystallization

The catalytic domains of native and mutant (E192Q) BoGT6a (residues 1–246) were expressed in *E. coli* BL21(DE3), purified as described previously (12), and stored at −4 °C in 20 mm Tris-HCl, pH 7, 100 mm NaCl, 2 mm DTT, 10 mm EDTA. The complexes of wild-type BoGT6a and of the BoGT6a E192Q mutant with UDP-GalNAc were prepared by mixing 190 μl of protein solution (8 mg/ml) with 10 μl of UDP-GalNAc (100 mm) and incubating at room temperature for 1 h or at 4 °C overnight. Crystallization screens were conducted using the sitting drop vapor diffusion method with a Phoenix crystallization robot on a 96-well Intelli-plate (Art Robbins Instruments). Designed crystallizations were set up manually using the vapor diffusion hanging drop method with 24-well plates. The drops were set up at a 1:1 ratio of protein to mother liquor and incubated at 16 °C. Crystals of the complex with UDP-GalNAc were obtained using the BoGT6a E192Q mutant, but none were obtained with the wild-type enzyme. One cluster of bar-shaped crystals was grown in a well solution containing 0.1 m sodium citrate, pH 5.0, 20% PEG 8000 with Proplex crystallization screen (Molecular Dimensions Ltd.). Many crystals appeared in two different solutions (namely 0.1 m Bis-Tris, pH 5.5, containing 0.2 m (NH_4_)_2_SO_4_ and 20% PEG 3350; and 0.1 m Bis-Tris, pH 5.5, containing 0.2 m Li_2_SO_4_ and 20% PEG 3350) on 24-well plates.

##### X-ray Data Collection and Processing

Diffraction datasets for the complex (to 3.50, 3.42, and 2.78 Å) were recorded at the Diamond Light source (Didcot, Oxon, UK) on stations I04 and I04-1 at 100 K. Cryo cooling was carried out prior to x-ray data collection, after stabilizing the crystals in 25% glycerol. The datasets were processed by Xia2 (14) in P2_1_ (*a* = 176.98, *b* = 79.77, *c* = 179.08Å, β = 95.2° for the 3.50 Å dataset) and P2_1_2_1_2_1_ (*a* = 80.12, *b* = 115.60, *c* = 126.12 Å for the 2.78 Å dataset and *a* = 80.12, *b* = 120.15, *c* = 131.83 Å for the 3.42 Å dataset).

##### Structure Determination

Phases for the BoGT6a E192Q/UDP-GalNAc complex at 2.78 Å (form I) were calculated using the native (apo) BoGT6a structure (13) as the starting model by the molecular replacement method using PHENIX software suite (15). The structure belongs to the orthorhombic space group P2_1_2_1_2_1_ and has four molecules in the asymmetric unit. The missing loop (from 126 to 150) in the original BoGT6a structure was built based on the electron density map. Because clear electron density was only observed for GalNAc, this ligand was inserted into the structure instead of UDP-GalNAc. Further refinement and model building were performed using the PHENIX software suite and COOT (15, 16). The structure of the BoGT6a E192Q-GalNAc complex was used as starting model for phase calculation of other complexes. In the second orthorhombic form (space group P2_1_2_1_2_1_ at 3.42 Å resolution, form II), there were four molecules per asymmetric unit, whereas a monoclinic form in space group P2_1_ (3.5 Å resolution, form III) contained 16 molecules per asymmetric unit. UDP-GalNAc, UDP, and GalNAc were selected for insertion into the protein structures based on their electron density. Despite low resolution, the high number of molecules in the asymmetric unit showed strong different electron densities for the N-terminal Met^1^ residue in some of the 16 chains, which was not observed in the other structures. In addition, two additional residues of the hexahistidine tag, namely His^0^ and Ser^−1^, were included because these were visible in some of the molecules. The final three structures were obtained through several refinement cycles using PHENIX and COOT.

## RESULTS AND DISCUSSION

**FIGURE 2.**
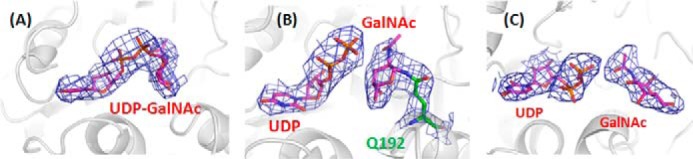
**Difference Fourier electron densities of ligands in the monoclinic structure.** *A*, intact UDP-GalNAc. *B*, proximal UDP and GalNAc. Clear electron density connects GalNAc and Gln^192^. *C*, hydrolysis products UDP and GalNAc. The map was contoured at 1 σ and is shown in *blue*.

Two orthorhombic crystal forms were obtained. One (form I) was grown in 0.1 m sodium citrate, pH 5.0, containing 20% PEG 8000 and diffracted at 2.78 Å, and the second (form II) was obtained in 0.2 m Li_2_SO_4_ in 0.1 m Bis-Tris, pH 5.5, containing 20% PEG 3350, and diffracted at 3.42 Å. Both orthorhombic forms had four molecules in the asymmetric unit, but electron density for only GalNAc was found in the form I structure. However, in the form II structure, two molecules (chains A and C) contain intact UDP-GalNAc and the others (chains B and D) contain the hydrolysis products, UDP and GalNAc; the GalNAc was located near the acceptor binding pocket, away from the position of the GalNAc in intact UDP-GalNAc. A monoclinic crystal form (form III) grew in 0.1 m Bis-Tris, pH 5.5, containing 0.2 m (NH_4_)_2_SO_4_ and 20% PEG 3350, and diffracted to 3.50 Å. This structure includes 16 protein molecules per asymmetric unit that contain three different ligand configurations clearly shown by their electron densities (Fig. 2). The crystallographic statistics for the three structures are summarized in Table 1.

**TABLE 1:** **X-ray crystallographic statistics**

	BoGT6a E192Q·GalNAc	BoGT6a E192Q·UDP-GalNAc	BoGT6a E192Q·UDP-GalNAc
Ligands used in crystallization	UDP-GalNAc	UDP-GalNAc	UDP-GalNAc
Ligands observed in crystal structure	GalNAc	UDP-GalNAc, UDP, GalNAc	UDP-GalNAc, UDP, GalNAc
Space group	P2_1_2_1_2_1_	P2_1_2_1_2_1_	P2_1_
No. of molecules/asymmetric unit	4	4	16
Cell dimensions	*a* = 80.12, *b* = 115.60, *c* = 126.12 Å	*a* = 80.12, *b* = 120.15, *c* = 131.83 Å	*a* = 176.98, *b* = 79.77, *c* = 179.08 Å, β = 95.23°
Resolution range (Å)	67.63–2.78	88.80–3.42	87.98–3.50
*R*_merge_ (outer shell)	0.10 (0.71)	0.09 (0.54)	0.13 (0.50)
I/σI (outer shell)	15.0 (2.3)	13.5 (2.8)	7.4 (2.1)
Completeness (outer shell) %	97.7 (99.5)	97.6 (99.8)	98 (99.0)
Total no. of reflections	158394	93516	168581
Unique no. of reflections	29475	17402	61949
Redundancy (outer shell)	5.4 (4.9)	5.4 (4.6)	2.7 (2.6)
Wilson B-factor (Å^2^)	45.73	93.61	76.41
*R*_cryst_/*R*f_ree_	23.14/27.35	28.35/31.41	22.53/24.94
Overall average B-factor (Å^2^)	41.19	82.25	71.38

**No. of protein chains**	4	4	16
UDP-GalNAc		2	6
UDP		2	10
GalNAc	4	2	10
Water	174		7
PEG			1
SO_4_^−^			1
Glycerol			5

**r.m.s.d.*^a^* values**			
Bond length (Å)	0.004	0.006	0.002
Bond angle (°)	0.979	1.206	0.529

**Ramachandran plot statistics (%)**			
Favored	96.15	92.71	93.66
Outliers	0.11	0.21	0.10

**RCSB-PDB codes**	4cjb	4cjc	4cj8

*^a^* r.m.s.d., root mean square deviation.

The recombinant BoGT6a construct used in this study lacks the C-terminal 17 residues of the wild-type BoGT6a, a region that has similar sequence characteristics to lipid binding domains in other proteins (6). This truncation reduces aggregation but does not affect catalytic activity (12). BoGT6a in these complexes has a similar overall structure to that reported for the apo-protein and its complex with FAL (13). As reported previously, BoGT6a has a GT-A fold similar to structures reported for bovine α3 GT (17) (Fig. 1) and histo-blood group A and B synthases (GTA and GTB) (18) but is shortened at the N terminus by 45 residues or more as compared with the mammalian GTs. The C-terminally truncated BoGT6a appears to represent the minimum size of a functional GT6 because residues close to both the N termini and the C termini participate in substrate binding. The structure comprises two contiguous subdomains: the N-subdomain (residues 1–97) that includes four parallel β-strands and three surrounding α-helices and the C-subdomain (residues 98–246) that includes a three-stranded mixed β-sheet and a small two-stranded β-sheets associated with two α-helices. In the mammalian GT6, the metal binding D*X*D motif is at the junction of the two subdomains but, in the bacterial enzyme, it is replaced by the Asn^95^-Ala-Asn^97^ sequence. The D*X*D motif is a shared feature of all GT families with GT-A folds (19, 20) with the exception of the metal-independent GT14 family (21).

### 

#### 

##### Interactions with Ligands and Conformational Changes

**FIGURE 3.**
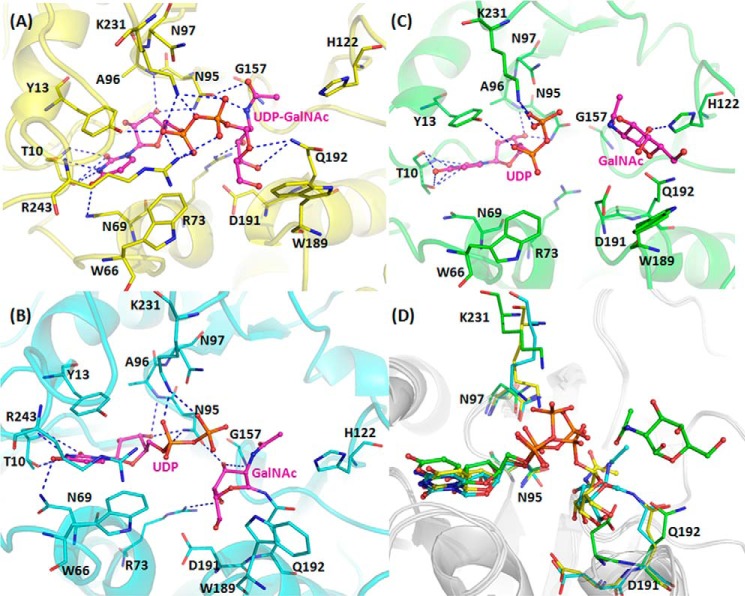
**Interactions of BoGT6a (E192Q) with bound ligands.** *A*, intact UDP-GalNAc (structure A). *B*, hydrolysis products, UDP and GalNAc, in close proximity (structure C). *C*, hydrolysis products, UDP and more distant GalNAc (structure B). Ligands are shown as sticks in *magenta*, and interacting residues are shown as lines. *D*, superimposition of three ligands, which are drawn as sticks, and key residues (Asn^95^, Asn^97^, Asp^191^, Gln^192^, and Lys^231^), shown as lines, colored to identify their chain: *yellow* for structure A, *green* for structure B, and *cyan* for structure C.

Although UDP-GalNAc was present in the crystallization solution, all molecules in form I contained only GalNAc. In contrast, in the second orthorhombic form (II) two chains, A and C, contained an intact UDP-GalNAc ligand, and the other two chains, B and D, contained the hydrolysis products, UDP and GalNAc. Therefore the E192Q mutant of BoGT6a had sufficient activity to hydrolyze significant amounts of substrate during the time taken to prepare the crystals. Form II crystals were obtained from the complex that had been incubated for 1 h, whereas the form I crystals that contain only GalNAc were grown from a preparation that had been incubated overnight, suggesting that the mutant GT had hydrolyzed most of the substrate and that the UDP product had dissociated from protein molecules in the crystals. Monoclinic form III was also prepared with protein that had been preincubated for only 1 h. It is less ordered than the form II structure and has 16 polypeptide chains in the asymmetric unit that comprise three groups. (i) Chains E, F, G, H, O, and P have intact UDP-GalNAc bound to a compact conformation similar to chains A and C of orthorhombic form II and designated structure A (Fig. 2*A*). (ii) Chains A, B, C, D, I, J, K, and L have the hydrolysis products, UDP and GalNAc, and a more open conformation similar to chains B and D of orthorhombic form II (designated structure B); in this group, the GalNAc is displaced and reoriented relative to that in UDP-GalNAc. (iii) chains M and N have hydrolysis products and a closed structure in which the GalNAc remains in a similar orientation and location to that in UDP-GalNAc (designated structure C) (Fig. 3, *A–C*). The relative arrangements of the ligands in the superimposed complexes are shown in Fig. 3*D*. No metal ions were identified in any of the structures.

**FIGURE 4.**
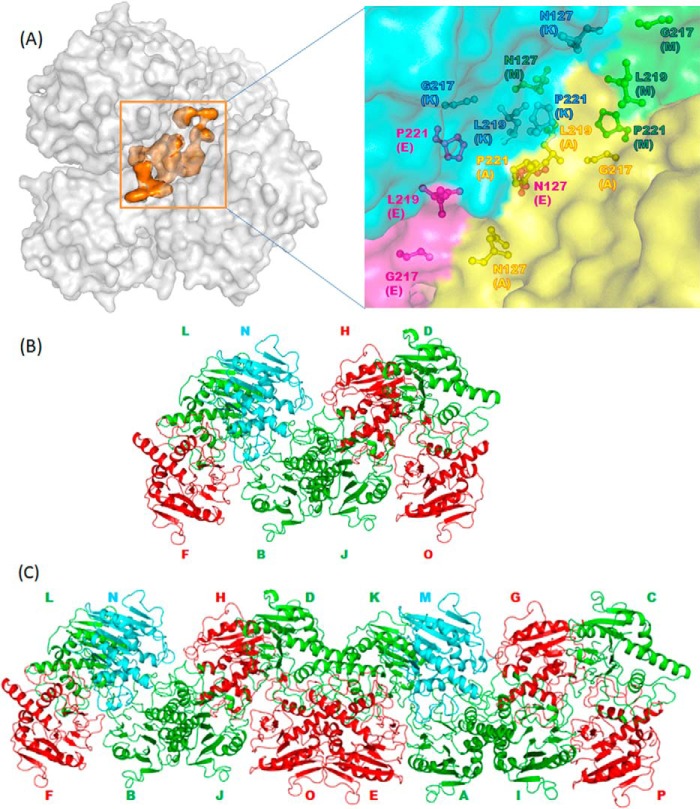
**Arrangements and interactions of BoGT6a molecules.** *A*, packing between chains in the monoclinic form III structure showing hydrophobic core interactions. The surfaces of protein molecules are colored *gray*, and the hydrophobic core is colored *orange*, respectively. The *inset* shows the details of the hydrophobic core in which the hydrophobic residues are displayed as sticks and colored by chains. *B*, arrangement of the minimal packing unit in the monoclinic crystal form. Structure A is in *green*; structure B is in *red*, structure C is in *cyan*, and protein molecules are shown as graphics. *C*, arrangement of 16 molecules in the asymmetric unit in monoclinic crystal form.

In all three crystal forms, the packing of molecules appears to be similar to that observed in the structure of BoGT6a in a complex with FAL (13). In form II, the interaction between each molecule with an intact substrate and each molecule with hydrolysis products is mediated by contacts between residues Asn^127^ and Leu^219^, and the symmetry between the two pairs is related by a pseudo translation. Each pair interacts with the other pair through Pro^221^-Gly^217^ and Glu^223^-Lys^233^ (Fig. 4*A*). Similar packing features are also observed in the monoclinic structure. However, this structure, with 16 molecules in the asymmetric unit, exhibits more elaborate packing. One chain with a compact structure containing UDP and GalNAc (structure C, *green*) leads to a group of two molecules with intact UDP-GalNAc (structure A, *red*), one molecule with an open structure containing UDP and GalNAc (structure B, *cyan*), and one with a compact structure containing UDP and GalNAc (Fig. 4*B*). Among the 16 molecules in the asymmetric unit, eight are related by pseudo translation (Fig. 4*C*). For discussion purposes, we have used representatives from each group, namely chain E for structure A, chain A for structure B, and chain M for structure C.

**FIGURE 5.**
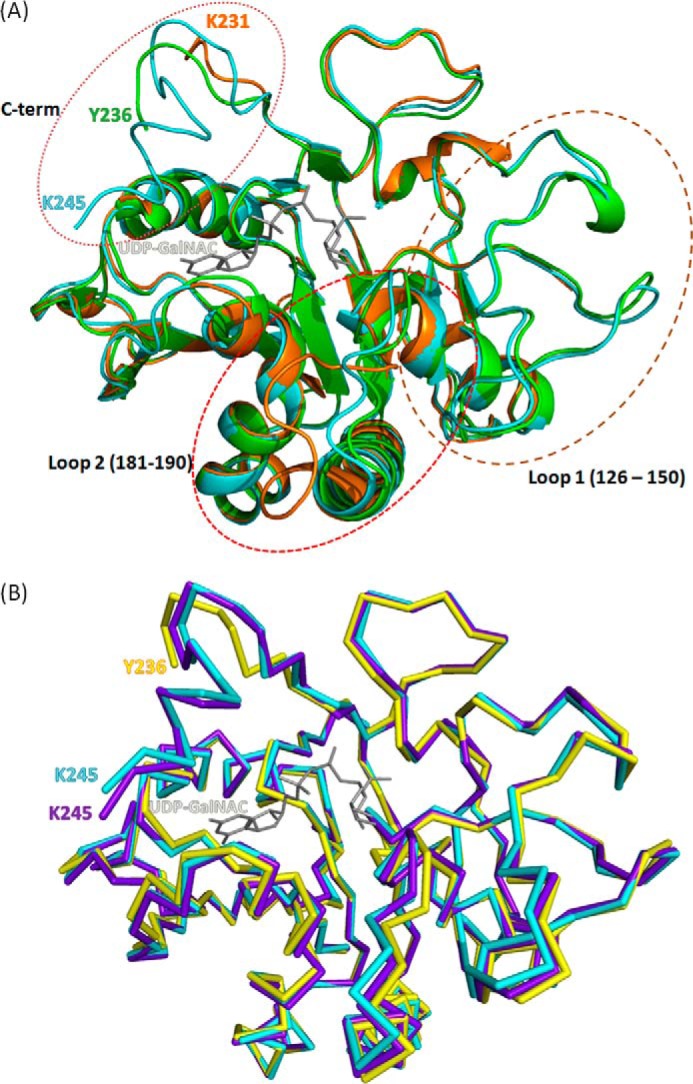
**Effects of binding different ligands on the conformation of BoGT6a.** *A*, superimposed ribbon structures of apo-protein (*orange*), FAL complex (*green*), and UDP-GalNAc complex (cyan). The UDP-GalNAc ligand from the latter complex is colored *gray*. The marked loops are regions that undergo major structural adjustments and are discussed under “Results and Discussion.” *B*, superimposed ribbon structures of chain A (structure B, *yellow*), chain E (structure A, *cyan*), and chain M (structure C, *purple*). The UDP-GalNAc ligand from chain E is colored *gray*.

**FIGURE 6.**
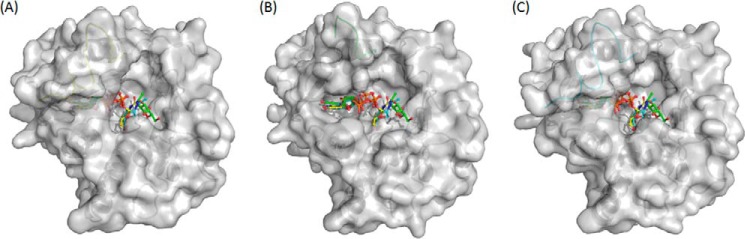
**Surface diagrams of three BoGT6a structures.** *A*, structure A. *B*, structure B. *C*, structure C. The protein surface is colored in *gray*, and C-terminal residues (229–245) and ligands are colored by chain structures (structure A in *yellow*, structure B in *green*, and structure C in *cyan*).

The binding sites for UDP-GalNAc and FAL are located in a pocket on the protein surface that, in the closed structure(s), is covered by a cap formed principally by restructuring the C terminus of the polypeptide chain. The conformation of the enzyme in all complexes containing ligands is more closed and more structured than the apo enzyme (Fig. 5*A*). The interaction with either substrate stabilizes loop 1 (residues 126–150), which is unstructured in the apo form (13), and induces the enzyme to undergo a conformational change to a less open form (Fig. 4*A*). In particular, in form II and form III, interactions with the donor substrate in the compact types of structure, A and C, stabilize part of the C terminus, and the structure can be followed beyond Lys^231^, which interacts with the diphosphate (Fig. 4*B*). However, there is some variation in the structure between different chains beyond this residue (for example between chains E, F, G, and H), suggesting that this region is flexible. In structures of type B, where the UDP-GalNAc has been hydrolyzed and GalNAc has moved to the acceptor binding site, there is greater flexibility than in structures A or C. In this state, its high level of mobility leads to an absence of electron density for the C-terminal 10 residues, and the models were built only up to residue 236. Similarly, in the complex of BoGT6a with the acceptor, loop 2 (residues 181–192) undergoes a significant conformational change that affects the positions of Trp^189^, His^190^, Asp^191^, and Gln (Glu)^192^, which are key residues for substrate binding and catalysis (Figs. 3 and 5*A*). Fig. 5*B* shows the superimposed structures A, B, and C (represented by chains E, A, and M); these show that the main conformational differences are in the C-terminal region of the structure. The surface diagrams of the three forms show that C (chain M) is slightly more open than A (chain E), whereas B (chain A) is the most open form in which both the UDP and the GalNAc ligands are exposed to solvent (Fig. 6). In the complexes with intact UDP-GalNAc, the residues that interact with different moieties of the substrate, are listed in Table 2.

**TABLE 2:** **Summary of all interactions between all chains of BoGT6a E192Q in monoclinic crystal form with ligands** Atom labels for the ligands are chosen according to Ligplot diagrams (as shown in Fig. 7). All interactions were analyzed using the PISA program (33).

Residue	Protein atom	Ligand atom		
		UD2	UDP	GalNAc
Thr^10^	[OG1]	[N3] [O2]	[N3] [O2] [O4]	X
	[N]	[O2]	[O2]	X
	[O]	[N3]	[N3]	X
Gln^192^	[N]	[O4′]	X	X
	[NE2]	[O4′] [O5′] [O6′]	X	[C1′] [O6′] [O5′] [O3′] [O4′] [O1′]
	[OE1]	X	X	[O6′]
Arg^73^	[NH2]	[O3′]	X	[O4′]
Gly^157^	[N]	[O3′]	X	X
Lys^231^	[NZ]	[O3A] [O1A] [O1B]	[O1A] [O2B] [O1B] [O2A] [O1′]	X
Tyr^13^	[OH]	[O1A] [O2A]	[O1A] [O2A] [O1B] [O2B] [O1′] [O3A]	X
Ala^96^	[N]	[O3B]	[O3B] [O2′]	X
Asn^95^	[ND2]	[O1A] [O3B][O5B]	[O3B] [O2B]	X
	[OD1]	[N2′]	X	X
Arg^243^	[NH2]	[O3A][O2A] [O2B] [O1A]	[O2A] (structure C)	X
Asn^69^	[ND2]	[O4]	[O4]	X
	[OD1]	X	[N3]	X
Trp^189^	[O]	[O6′]	X	X
His^239^	[NE2]	X	[O2B]	X
Trp^66^	[NE1]	[O2A]	[O2B]	X
Thr^70^	[OG1]	X	[O1′]	X
His^190^	[N]	X	X	[O6′]
Asp^191^	[N]	X	X	[O6′]
Thr^134^	[OG1]	X	X	[O6′]
His^122^	[NE2]	X	X	[O4′] [O1′]

**FIGURE 7.**
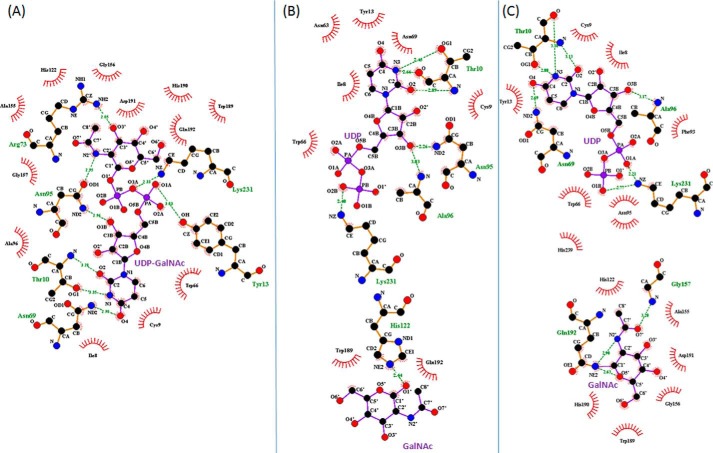
**Ligplot of BoGT6a E192Q-ligand interactions with key residues in different complexes (32).** *A*, structure A, *B*, structure B. *C*, structure C. Ligands are shown in *purple*, interacting residues are shown in *orange*, and hydrophobic interacting residues are shown in *red*. Hydrogen bonds are shown as *green dashes*.

Although Asn^95^ has multiple interactions with UDP-GalNAc, Asn^97^, the second Asn of the N*X*N motif, does not interact with the substrate. A superimposition of the intact substrate in chain E (structure A) and the hydrolysis products in chain A (structure B) and M (structure C) shows that the uracil and ribose are positioned similarly in all three structures (Fig. 3*D*), and there are similar interactions between the uracil and ribose moieties and protein in the structures, but those involving the diphosphate and GalNAc differ. In structure A (intact UDP-GalNAc), the ϵ-amino group of Lys^231^ H-bonds with O1A, O1B, and O3A atoms, and the NH_2_ of Arg^243^ interacts weakly with different oxygens in different chains. Also the OH of Tyr^13^ and peptide N of Ala^96^ H-bond with the diphosphate (Fig. 7). In structure C, the amino group of Lys^231^ interacts more strongly with O1A and also with O1B of the diphosphate, and the NH_2_ of Arg^243^ is near to but not in H-bonding distance (3.66 Å) of O2A. Ala^96^ interacts with O3B, but there is a stacking interaction between Tyr^13^ and the substrate. In structure B, although there are similar interactions between the protein and the uracil and ribose, the diphosphate has a variable orientation in different chains, and the Lys^231^ NH_2_ and Tyr^13^ OH interact with different oxygens, although the Ala^96^ NH and Asn^95^ ND2 mainly interact with O3B. The GalNAc in structure A interacts with Gln^192^ NE2 through O4′ and Arg^73^ NH_2_ and Gly^157^ N through O3′ as well as with Asn^95^ OD1 through N2′. In structure C, the β-GalNAc C1 is in close contact with Gln^192^ NE2 and with Arg^73^ NH_2_ through O4′. In structure B, where the GalNAc is located in the acceptor binding site, there are no consistent interactions with the protein in different chains.

The A structure represents the UDP-GalNAc Michaelis complex with BoGT6a, but it should be noted that the Glu^192^ to Gln mutation in the form of BoGT6a used in these structural studies involves a key residue in catalysis (12), which interacts with both the donor and the acceptor substrates (13). Previous isothermal titration calorimetry studies have shown that this mutation has little net effect on the Δ*G* for UDP-GalNAc binding (both about −5.8 kcal/mol), but this reflects mutually compensating effects on the Δ*H* and *T*Δ*S* of binding (changes from −23.5 to −16.4 kcal/mol and 17.7 to 10.6 kcal/mol, respectively), suggesting that the mutation may weaken noncovalent interactions and reduce substrate-induced conformational rearrangements (13). Structures A, C, and B are models for complexes in the hydrolysis reaction catalyzed by BoGT6a, -C, and -B, being earlier and later steps in product release. However, it should be recognized that these are structures stabilized by being incorporated in different locations in the unusual monoclinic crystal lattice. Only structures A and B are present in molecules in the orthorhombic form.

Although the structure of a ternary complex of BoGT6a with both UDP-GalNAc and FAL has not been determined, examination of the structures of the complexes with individual substrates indicates that the UDP-GalNAc binding site is not accessible in the FAL complex (13) but the FAL binding site is accessible in the complex with UDP-GalNAc. In a modeled ternary complex generated from the complexes with individual substrates, it can be seen that UDP-GalNAc is bound deeply in the structure and that the FAL molecule hinders its solvent access and release. The structures therefore suggest that BoGT6a utilizes an ordered sequential mechanism in which the donor substrate binds prior to acceptor. This is consistent with isothermal titration calorimetry studies that show FAL binds weakly to free BoGT6a (*K_d_* 1.2 mm) but more strongly to the UDP complex (*K_d_* 76 μm); the change in free energy of binding (−1.64 kcal/mol) arises from a more favorable enthalpy of binding (δΔ*H* of −1.9 kcal/mol).

##### Structure-Function Relationships in Metal-dependent and Metal-independent GT6

Previously, crystallographic and mutational studies of structure-function relationships have been conducted with the catalytic domains of mammalian metal-dependent GT6, bovine α3GT (17, 22–25), and human GTA/GTB (18, 26–28). In the complexes containing Mn^2+^ and UDP or a UDP derivative, the metal interacts with the aspartates of the D*X*D motif (residues Asp^225^-Val-Asp^227^ in α3GT), through a single interaction with Asp^225^ and a bidentate interaction with Asp^227^, and also interacts with an oxygen from each of the α- and β-phosphates of the UDP (17, 18). Val^226^ and Asp^227^ also interact with the ribose moiety of the UDP moiety. In complexes containing free UDP, two cationic residues close to the C terminus of α3GT, Lys^359^ and Arg^365^, interact with the diphosphate, but in the complex of the low activity mutant of α3GT (E317Q) with the substrate UDP-gal, the side chain of Lys^359^ is disordered and only Arg^365^ interacts with the α-phosphate (22). The interaction with Arg^365^ is facilitated by structural changes in a loop containing Trp^195^, with which Arg^365^ forms a stacking interaction. In the structure of a complex of the inhibitory substrate analog, UDP-2F-gal, with the R365K mutant of α-3GT, the side chain of Lys^359^ points toward the β-phosphate of the inhibitor, but the C-terminal 9 residues including Arg^365^ are disordered (23). It should be noted that these complexes contain catalytically impaired mutants of the enzyme and, in one case, an inhibitor rather than substrate and are imperfect models of the enzyme-substrate complex. Both Lys^359^ and Arg^365^ make contacts with UDP in its complex with α3GT (22, 23), and conservative substitutions of Lys^359^ to Arg or Arg^365^ to Lys result in >30-fold reductions in *k*_cat_ but have small (∼2-fold) effects on the *K_m_* for UDP-gal (23); the Lys to Ala mutation produces a 350-fold reduction in *k*_cat_. Therefore Lys^359^ and Arg^365^ are important, although not essential, for activity and appear to have a principal role in transition state stabilization, mediated through interactions with the UDP leaving group, as opposed to (ground-state) substrate binding.

Lys^231^ of BoGT6a is homologous with Lys^359^ of α3GT, a residue that is conserved in all metal-dependent (D*X*D) and metal-independent (N*X*N) GT6. However, the residue corresponding to Arg^365^ of α3GT is conserved in most other metal-dependent GT6 enzymes but not in the bacterial enzymes. The present structural studies indicate that Arg^243^ of BoGT6a has a similar structural location to α3GT Arg^365^ but appears to interact less specifically with the diphosphate moiety. Also, it is not within H-bonding distance of the diphosphate in structure C. Substitution of Lys^231^ by Ala increased the *K_m_* about 2-fold but reduced *k*_cat_ more than 200-fold, whereas the double mutation of Arg^243^ and Arg^244^ to Ala reduced *k*_cat_ by a factor of 10 (12). Thus, Lys^231^ has a greater stabilizing effect on the transition state than ground state and appears to help to stabilize the UDP leaving group during catalysis, a role similar to that of the homologous Lys in α3GT, which is consistent with the present structural studies.

##### Other Metal-independent Members of the GT-A Superfamily

The majority of Leloir GTs, enzymes that utilize sugar nucleotides as donor substrates, group into either of two large superfamilies that have GT-A and GT-B folds. The GT-B superfamily is metal-independent, whereas most representatives of the GT-A fold superfamily that have been functionally characterized are metal-dependent and have D*X*D metal binding motifs (4). The sialyltransferases, whose donor substrate is a nucleotide monophosphate sugar (CMP-sialic acid), are metal-independent and include proteins with GT-A and GT-B-like folds, but with distinct topologies; comparisons of structure-function relationships between sialyltransferases and other GTs are challenging because of their nonstandard folds and the character of the donor substrate (29). Among the members of the “standard” GT-A group that have been functionally characterized, only the members of GT14 are metal-independent like BoGT6a. The GT14 and GT6 families differ in catalyzing inverting and retaining reactions, respectively, but GT14 members also differ in having no conserved motif corresponding to D*X*D (or N*X*N). The crystallographic structures of one GT14, leukocyte type core 2 β1,6-*N*-acetylglucosaminyl-transferase (C2GnT-L), in the apo-form and in complexes with either acceptor substrate or UDP, have been determined (21, 30). The enzyme is a disulfide-bonded dimer and, in the complex with UDP, the two molecules of each dimer are in “open” and “closed” conformations. In the closed conformation, two basic residues close to the C terminus of the protein, Arg^378^ and Lys^401^, interact with the β-phosphate of the UDP, but these interactions are not present in the open conformer. Substitution of either Arg^378^ or Lys^401^ by Ala eliminates catalytic activity, but the Arg^378^ mutation has relatively small effects on the binding of UDP or UDP-GalNAc, whereas the Lys^401^ mutation eliminates binding. Based on these observations, these two cationic residues were proposed to fulfill the role of the metal ion in metal-dependent GT-A GTs. Although the characterized enzymes of the GT14 family are metal-independent, in some cases, the activity is enhanced by divalent metals (31). Therefore they appear to have a metal binding site and may have evolved from a metal-dependent ancestor. In contrast, the activity of BoGT6a decreases when increasing levels of Mn^2+^ ion are added to the enzyme (12).

##### Conclusion

As discussed above, two C-terminal basic residues in BoGT6a interact with the donor substrate, but these interactions are similar to those in metal-dependent GT6. If we surmise that the GT-A superfamily evolved from a metal-dependent common ancestor, it would seem that the GT6 and GT14 families have used different adaptations to become metal-independent. In the GT6 family, we propose that the replacement of the D*X*D motif by N*X*N was a major factor in the transition between metal dependence and metal independence. This double substitution removes the requirement for a divalent metal ion to counter charge repulsion between the aspartates and diphosphate of the UDP. Therefore in the metal-dependent GT6, the role of the metal ion in donor substrate binding in the ground state and in stabilizing the UDP leaving group in the transition state (4) is effectively performed by the polypeptide in the metal-independent GT6.

## Footnotes

6 The abbreviations used are:

GT: glycosyltransferase
α3GT: α-1,3-galactosyltransferase
HBG: histo-blood group
FAL: 2′-fucosyl-lactose
Bis-Tris: 2-(bis(2-hydroxyethyl)amino)-2-(hydroxymethyl)propane-1,3-diol.
